# Molecular Detection of a Potentially Toxic Diatom Species

**DOI:** 10.3390/ijerph120504921

**Published:** 2015-05-06

**Authors:** Bidhan Chandra Dhar, Lucia Cimarelli, Kumar Saurabh Singh, Letizia Brandi, Anna Brandi, Camilla Puccinelli, Stefania Marcheggiani, Roberto Spurio

**Affiliations:** 1 Laboratory of Genetics, School of Biosciences and Veterinary Medicine, University of Camerino, Camerino 62032, Italy; E-Mails: bidhan.dhar@gmail.com (B.C.D.); lucia.cimarelli@studenti.unicam.it (L.C.); kumar.saurabhsingh@unicam.it (K.S.S.); letizia.brandi@unicam.it (L.B.); anna.brandi@unicam.it (A.B.); 2 Environmental, Quality and Fishfarm Unit, Environment & Primary Prevention Department, Istituto Superiore di Sanità, Viale Regina Elena, Rome 299, Italy; E-Mails: camilla.puccinelli@iss.it (C.P.); stefania.marcheggiani@iss.it (S.M.)

**Keywords:** diatoms, domoic acid, oligonucleotide probes, microarrays

## Abstract

A few diatom species produce toxins that affect human and animal health. Among these, members of the *Pseudo-nitzschia* genus were the first diatoms unambiguously identified as producer of domoic acid, a neurotoxin affecting molluscan shell-fish, birds, marine mammals, and humans. Evidence exists indicating the involvement of another diatom genus, *Amphora,* as a potential producer of domoic acid. We present a strategy for the detection of the diatom species *Amphora coffeaeformis* based on the development of species-specific oligonucleotide probes and their application in microarray hybridization experiments. This approach is based on the use of two marker genes highly conserved in all diatoms, but endowed with sufficient genetic divergence to discriminate diatoms at the species level. A region of approximately 450 bp of these previously unexplored marker genes, coding for elongation factor 1-a (eEF1-a) and silicic acid transporter (*SIT*), was used to design oligonucleotide probes that were tested for specificity in combination with the corresponding fluorescently labeled DNA targets. The results presented in this work suggest a possible use of this DNA chip technology for the selective detection of *A. coffeaeformis* in environmental settings where the presence of this potential toxin producer may represent a threat to human and animal health. In addition, the same basic approach can be adapted to a wider range of diatoms for the simultaneous detection of microorganisms used as biomarkers of different water quality levels.

## 1. Introduction

Diatoms are single-celled photoautotrophic microorganisms present in virtually any habitat, though they are principally found in association with submerged surfaces (benthic) or suspended in the water column of rivers, lakes, estuaries and oceans (planktonic). In addition to their contribution to net primary production, these algae can produce extracellular polymeric substances and storage polysaccharides (e.g., chrysolaminarin) and can accumulate a range of bioactive metabolites, such as PUFAs (poly-unsaturated fatty acids), which are an excellent food source for invertebrate grazers and harmful biotoxins [1,2,3]. The first severe intoxication case caused by diatoms was described in 1987, when three people died and over 100 became ill after eating cultured mussels from Prince Edward Island, Canada [4,5,6]. A careful examination of the shellfish responsible for this intoxication, revealed the presence of high amounts of a potent glutamate agonist: the toxin domoic acid (DA). Domoic acid is a secondary metabolite structurally similar to kainic acid and the amino acids glutamic acid and aspartic acid. DA is responsible for Amnesic Shellfish Poisoning (ASP), whose effects in humans include vomiting, abdominal cramps, diarrhea, headache up to permanent short-term memory loss, coma, and death. Multiple events of ASP have occurred in sea lions, seabirds, fish and marine mammals [7,8]. Domoic acid accumulates in filter-feeding shellfish following consumption of toxin producing phytoplankton. The diatoms most frequently found associated with domoic acid production belong to the *Pseudo-nitzschia* genus, harmful algae isolated from many coastal and estuarine waters, among which those of United States and Canada [9], Portugal [10], Spain [11], France [12], Italy [13,14], Croatia [15], Greece [16], Ireland [17] and Australia [18,19].

In addition to *Pseudo-nitzschia*, a second diatom genus (*Amphora*) has been described as a DA producer [6]. A small pennate marine diatom was in fact recognized as a source of DA in the contaminated Canadian mussels by Shimizu *et al.* [20] and Maranda *et al.* [21] and subsequently it was identified as *Amphora coffeaeformis* (Agardh) Kützing by Paul Hargraves of the University of Rhode Island (USA). The presence of *Amphora coffeaeformis* has also been reported in marine and inland waters collected from Argentina. Based on a detailed morphological analyses carried out by scanning electron microscopy, the authors demonstrated how frequently this diatom has been erroneously confused with other diatom taxa and questioned the identification of the Canadian *Amphor*a clone [22].

The genus *Amphora* Ehrenberg ex Kützing *sensu lato* is a wide group of benthic diatoms distributed in fresh, brackish and marine waters, with a predominance of species in marine environments [23,24]. There are more than 800 species of *Amphora* *sensu lato* described, with a taxonomic classification that is under continual revision [25,26,27]. With the increased use of scanning electron microscopy (SEM) observations, taxa within *Amphora* *sensu lato* have been transferred to several other genera, including the recent elevation of the *Amphora* subgenus *Halamphora* to level of genus [26]. In this classification, 79 new taxa from the genera *Amphora* and *Halamphora*, 63 of which were collected from freshwater environments, have been described. More recently, Stepanek *et al.* [28] described three new diatom species from the genus *Amphora* *sensu stricto* and four species from the genus *Halamphora*, collected from inland waters of USA. In addition, eight diatom species of *Amphora* *sensu lato* were reported from Korean coastal waters and evaluated in the context of morphological characteristics as well as of the phylogenetic position [29].

Because of the intense scientific and economic interests in detecting and monitoring environmental presence of toxin producers, several molecular methods have been developed for the early detection of blooms and prediction of toxin accumulation. Species-specific DNA probes directed toward nuclear-encoded ribosomal RNA (rRNA) were used to discriminate between toxic and nontoxic species of *Pseudo-nitzschia* in whole-cell and in sandwiches hybridization techniques [30] and recently quantitative real-time PCR was applied as an efficient molecular tool for the detection of phytoplankton cells at the pre-bloom levels [31]. Of the approximately forty species identified worldwide as *Pseudo-nitzschia* genus, fourteen have been confirmed as potential DA producers, but a more thorough investigation is required to establish the relationship between individual *Pseudo-nitzschia* strains, DA synthesis, and the influence of environmental factors on toxin production [32,33,34,35]. This certainly holds true also for *A. coffeaeformis*, for which no data are available on the physical/chemical parameters triggering the synthesis of domoic acid*.*

Despite the absence of reported cases of ASP in humans since 1987, the presence of phytoplankton producing biotoxins is routinely monitored in several coastal areas worldwide because of its relevant negative impact on human health, marine environment, and related economic activities. In connection to this, in 2001 a workshop named LIFEHAB [36] was organized in Spain by European Commission as a forum of discussion among specialists of different fields (taxonomy, physiology, ecology, molecular biology, and modeling) and of different harmful microalgae (diatoms, dinoflagellates, prymnesiophytes, and raphidophytes) with the aim of summarizing the knowledge available on life history of harmful algal blooms (HAB). The impact of DA on human health has been widely discussed in a review article [37]. The authors highlighted the effects of long-term exposure on individuals taking up low levels of DA from contaminated shellfish and stressed the need for rapid and effective monitoring of HAB events.

The present study focuses on the development of a strategy based on molecular methods, for the detection of *Amphora coffeaeformis* (Agardh) Kützing, a potential producer of the neurotoxin domoic acid. Following isolation of this diatom from estuarine waters collected from an estuarine setting on the Tyrrhenian coast of Central Italy and its successful cultivation under laboratory conditions, we sought to devise a molecular method for monitoring the presence of this biotoxin producer using a technology requiring less expertise than the traditional microscopy-based approach. Using a combination of species-specific oligonucleotide probe design and microarray analysis, we have set-up a reliable and sensitive methodology for the recognition of a potentially harmful diatom that may affect, through contamination of the food chain, both human and animal health.

## 2. Materials and Methods

### 2.1. Diatom Cell Cultures and Identification

**Figure 1:**
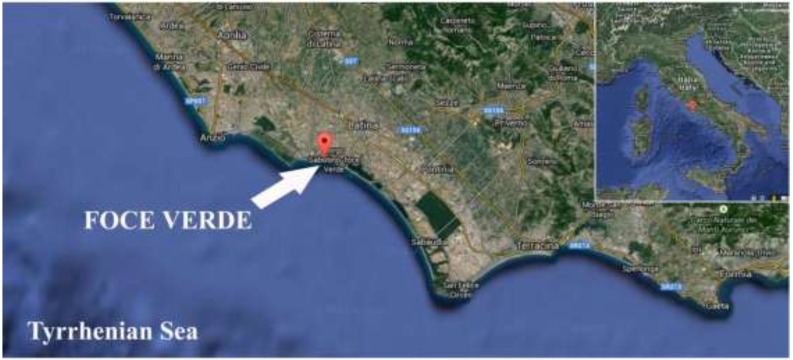
Map of the sampling site along Tyrrhenian coast of Lazio Region; The white arrow indicates the site of Foce Verde (41°24.937'N Latitude; 12°48.755'E Longitude) where the estuarine water was collected.

Environmental samples for this study were collected from estuarine waters located at Foce Verde, a channel located on the Tyrrhenian coast of Lazio Region, Italy (Figure 1) and were processed at the Laboratory of Genetics of the University of Camerino (Camerino, Italy).

Manual isolation of single diatom cells from cell suspension was achieved using a micropipette under a ZEISS Axiovert 25 inverted microscope at 20× magnification. Twenty-four isolated single diatom cells were placed in a multi-well plate and were repeatedly washed in sterile distilled water. They were cultured in Artificial Seawater Medium (ASW) [38,39], containing 24.5 g/L NaCl. The cultures were maintained at 18 °C under 12 h/12 h dark/light cycle. During the incubation period, the unidentified species were monitored daily checking cell density using a hemocytometer counting chamber (Bürker chamber, Saaringia—Germany) and once the culture reached the exponential growth phase, it was prepared for taxonomic identification. Culture identification was performed by experts at the Italian National Institute of Health (Rome), by treating cells with oxidizing agents as described [40], followed by mounting the diatom frustules deprived of organic substances on Naphrax^®^ slides, which were subsequently analyzed for species level determination using an Optiphot-2 microscope (Nikon, Japan) at 1000× magnification. In addition, photographs of live diatom cultures were taken and compared with images present in the reference iconographic guide [41]. Following morphological identification, the pure culture of *Amphora coffeaeformis* was maintained at the Culture Collection of the Laboratory of Genetics of the University of Camerino (Camerino, Italy).

### 2.2. Preparation of Genomic DNA

Cultures of *Amphora coffeaeformis* cells were propagated in 600 mL of ASW medium and after a period of three to four weeks of incubation were harvested by filtration on 0.45 μm Millipore filter. Chromosomal DNA was extracted from pure cultures of diatoms following an optimized protocol developed in our laboratory [42]. An additional step, consisting of incubation for 40 min at 37 °C with Proteinase K immediately after RNase treatment, was introduced during the DNA preparation. All the DNA samples isolated in this study were stored in the Diatom DNA Bank at the laboratory of Genetics of the University of Camerino, (Camerino, Italy).

### 2.3. Primer Design and Oligonucleotide Probe Design

All the DNA sequences were retrieved from Genbank and aligned using Clustal W (http://www.ebi.ac.uk/Tools/msa/clustalw2/). The sequences were neither trimmed nor edited. The conserved regions of the two marker genes, deduced from the sequence alignment, were used to locate primers for DNA amplification using OligoCalc software (http://www.basic.northwestern.edu/biotools/oligocalc.html) and a manual checking procedure. Individual stretches of oligonucleotides ranging from 25 to 35 bp were designed and experimentally tested in Polymerase Chain Reaction (PCR) based assays for their ability to amplify the target genes from all the chromosomal DNA of the Diatom DNA bank of our laboratory. All the species-specific oligonucleotide probes developed in this study were designed using combination of tools like Clustal W, BLAST, Primer3, Primer-BLAST and OligoCalc. The amplified regions obtained with the universal primers, were subjected to BLAST search. The resulting hits were then aligned to see if any subset of alignment produced species-specific regions. These potential specific regions were checked for T_m_ and secondary structures and blasted again to confirm the production of hits for the diatom species of interest. Only the top three hits were chosen for the synthesis of oligonucleotide probes used in microarray experiments.

### 2.4. DNA Amplification of Marker Genes from Genomic DNA

PCR reactions were performed in a T-Gradient DNA thermal cycler (Biometra^®^, Göttingen, Germany). Each reaction mixture contained: 1× *Pfu* buffer (20 mM Tris-HCl, 10 mM (NH_4_)_2_SO_4_, 10 mMKCl, 0.1% Triton X-100, 0.1 mg/mL BSA, 2 mM MgSO_4_, pH 8.8), 150 μM dNTPs, 0.4 μM of each primer, 0.5X Denhardt’s solution (1% Ficoll, type 400), 1% polyvinyl pyrrolidone, and 1% bovine serum albumin), and either *Pfu* DNA polymerase (Fermentas-Thermo Fisher Scientific, Waltham, MA, USA) or EconoTaq (Lucigen Corporation, Middleton, WI, USA). The amount of template used, consisting of purified genomic DNA extracted by CTAB method, was empirically optimized by performing a series of pilot reactions with amounts of DNA in the range 2–20 ng.

The primer pair UniE-F/UniE-R and the primer pair UniS-F_1_/UniS-R_1_ were used for amplification of Elongation Factor Gene (eEF1-a) and for amplification of silicic acid transporter gene (*SIT*), respectively (Table 1).

**Table 1:** Universal primers for DNA amplification of genomic fragments of eEF1-a and *SIT* genes.

Target Gene	Primer	Source	Nucleotide Sequence (5’-3’)	Amplicon Size (bp)
SSU rRNA	528F	[42]	GCGGTAATTCCAGCTCCAA	190
	650R	[42]	AACACTCTAATTTTTTCACAG	
Elongation Factor gene (eEF1-a)	UniE-F	This work	ATCGAACAACACAAGAAGGT	442
	UniE-R	This work	ACCTTTCCAAGCATCTTCAA	
Silicic acid Transporter gene (*SIT*)	UniS-F_1_	This work	GACTTCATCAACAACTACTTCG	474
	UniS-R_1_	This work	ACGTCCAATCATGAATCCAG	

The efficiency of DNA amplification was monitored by loading 5 μL of reaction on 2% agarose gel in TBE buffer (50 mM Tris-HCl pH 8.0, 50 mM Boric acid, 1 mM EDTA). The experimental conditions used to amplify DNA fragments of the two marker genes from genomic DNA are reported in Table 2.

**Table 2:** Polymerase Chain Reaction conditions used to amplify selected fragments of elongation factor gene (eEF1-a) and silicic acid transporter gene (*SIT*).

Target	Primers	Initial Denaturation		Denaturation		Annealing		Extension		Final extension		N° of Cycles
		T (°C)	Time (min)	T (°C)	Time (sec)	T (°C)	Time (Sec)	T(°C)	Time (sec)	T (°C)	Time (min)	
eEF1-a	UniE-F/UniE-R	95	2.5	94	30	58	60	72	30	72	2	24–30
*SIT*	UniS-F_1_/UniS-R_1_		3		30	50–58	40–50		40		2	30

The amplified DNA fragments were purified using the PCR Clean-up KIT (Sigma-Aldrich, St. Louis, Missouri, USA) to remove enzyme, nucleotides and primers according to the manufacturer’s protocol. When required, the amplicons were purified by extraction from agarose gel using GenElute Gel Extraction Kit (Sigma-Aldrich, St. Louis, Missouri, USA). After assessment of the concentration by NanoDrop^®^ ND-1000 (ThermoFisher Scientific, Waltham, Massachusetts, USA), an aliquot of the amplicon (approximately 80 ng) was used to determine the DNA sequence by dideoxynucleotide sequencing, while the remaining material was stored for subsequent use in hybridization experiments on microarrays.

### 2.5. Set-Up of the Microarray Format

Each oligonucleotide probe consists of a DNA sequence complementary to a sequence provided by the DNA target, and a spacer located at the 5’ end. The presence of a spacer, consisting of a poly (T_15_) tail, contributes to raising the spotted probes above the surface of the glass slide, and making them more accessible for hybridization thereby resulting in an enhanced fluorescent signal.

**Figure 2:**
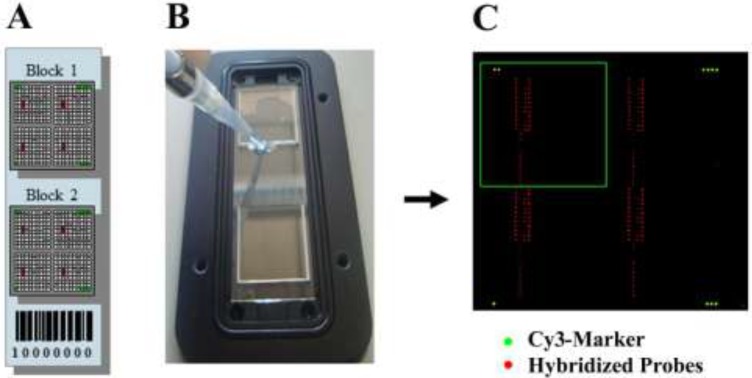
(**A**) Schematic overview of a microarray slide consisting of two blocks. Each block has four identical grids of probes; (**B**) Glass slide during loading procedure of a hybridization mixture; (**C**) Visualization of an hybridized slide after scanning: the green spots indicate the Cy3-Marker spotted at the four corners of the slide; the red spots indicate the positions where a fluorescent fragment of *Amphora coffeaeformis SIT* gene recognized the complementary oligonucleotide probes; the green square indicates one of the four sectors of one block.

As indicated in Figure 2, each epoxy-coated glass slide contained 2 blocks of oligonucleotides and each probe was spotted in eight replicas.

A control probe was introduced in the DNA chip to monitor the efficiency of hybridization. This probe is complementary to a fragment (250 bp) of the TBP control (TATA box Binding Protein) from the yeast *Saccharomyces cerevisiae*. The fluorescent TBP target was prepared by amplification of DNA extracted from bread yeast powder with primers TBP-F (5’-ATGGCCGATGAGGAACGTTTAA) and TBP-R (5’-Cy5-TTTTCAGATCTAACCTGCACCC). Five ng of purified TBP control were added to each hybridization mixture. Fluorescent signals of the control probes provide a measure of the hybridization efficiency and are used to normalize data within the hybridized array and between hybridization results obtained with different samples.

### 2.6. Microarray Experiments

Microarray experiments were performed at Scienion AG (Berlin, Germany) following a protocol developed *ad hoc* for the analysis of environmental water samples. The procedure consists of a series of steps:

Step (1): printing of DNA microchip. Oligonucleotide probes were printed on glass slides using equipment and technologies produced by the specialized company (Scienion AG). The relatively low number of species represented on this array allows each spot to be replicated 8 times in each of the two blocks, increasing the accuracy of measurement (red spots in Figure 2A). Two different microarrays were used in this study: MicroAqua-array-01 consisting of 174 oligonucleotide probes (Figure S1) for hybridization with elongation factor eEF1-a gene fragment and MicroAqua-array-02 consisting of 131 oligonucleotide probes (Figure S2) for hybridization with silicic acid transporter gene fragment (*SIT*). The sequences of oligonucleotide probes targeting *Amphora coffeaeformis* are listed in Supplementary materials (Table S1).

Step (2): labeling of diatom DNA sample. DNA samples were labeled using a Platinum *Bright* ^TM^ 647 Infrared Nucleic Acid Labeling Kit (KREATECH Diagnostics-The Netherlands), based on formation of a platinum complex with a fluorophore molecule, called Universal Linkage System (ULS), that labels DNA by binding to the N^7^ position of guanines. 200 ng of diatom DNA were used for the labeling reaction. DNA was initially denatured for 3 min at 95 °C and subsequently incubated with an hybridization mix (final volume 20 μL). The mix contained 1 μL of KREATECH ULS dye and 2 μL of 10× Labeling solution provided by the manufacturer. Following incubation for 1 h at 85 °C in a PCR thermocycler, DNA samples were placed on ice for 5 min before proceeding with purification.

Step (3): purification of labeled sample. To remove unreacted reagent, the labeled samples were added directly onto KREA*pure*^TM^ columns (KREATECH Diagnostics-The Netherlands) and centrifuged for 1 min at full speed. The purified labeled sample was recovered in the flow-through fraction.

Step (4): calculation of the degree of labeling. A spectrophotometric analysis was carried out by reading absorbance at 260 nm and 650 nm wavelengths and from those values was determined the Degree of Labeling (DoL) using the equation:

$$DoL=\frac{340\times 4}{1000\times 0}\;0000\%$$

where,

$$\text{X}=\text{ ng }\left(\text{nucleic acid}\right)/\text{µL }=\frac{\left(\text{OD}260–\left(\text{OD max }\times \text{ correction factor}\right)\;\right)\times \text{ dilution factor }\times \;40}{\text{cuvette path length }\left(\text{cm}\right)}$$

$$\text{Y}=\text{ pmol }\left(\text{dye}\right)/\text{µL }=\frac{\text{OD }\left(\text{dye}\right)\;\times \text{ dilution factor}}{\text{cuvette path length }\left(\text{cm}\right)\;\times \text{ ε dye }\times 1\times 10^{-6}}$$

The correction factor value = 0.05 and the ε dye = 250,000 mol^−1^ × L × cm^−1^ were used for ULS-Cy5, the fluorescent dye used in this study. Labeling was considered successful if the DoL value was between 1.0 and 3.0.

Step (5): hybridization. The hybridization reaction contained, in the initial volume of 45 μL, 12 μL of 5× hybridization buffer (2.5 mg/mL BSA, 0.5 μg/μL salmon sperm DNA, 5 M NaCl, 50 mM Tris-HCl pH 8.0, 0.025% Triton X-100), 5 ng of TBP control, Poly-dA (final concentration 50 nM), 6 μL of formamide (final concentration 10%), and 20 μL of labeled DNA sample. A fragment of the gene coding for TBP of *Saccharomyces cerevisiae* was amplified by PCR, labeled and used in the hybridization mixture as internal positive control. After initial denaturation for 5 min at 95 °C, the hybridization mixture was placed immediately on ice and mixed with 15 μL of KREA*block* (KREATECH Diagnostics-The Netherlands), a background blocker that decreases the background level and increases the hybridization efficiency. During this step, glass slides were covered with cover slips (22 × 22 mm, m Series Lifter Slips, Erie Scientific) and placed into the hybridization chamber. A half volume of denatured hybridization solution was added to each of the two arrays of the slide, at the rim of the cover slip, then the hybridization chamber was tightly closed with a glass lid and incubated in a water bath for 1 h at 55 °C.

Step (6): washing. This step was carried out in three separate wash vessels containing 200 mL of Wash Buffer 1 (2× SSC, 10mM EDTA pH 8.0, 0.05% SDS), Wash Buffer 2 (0.5× SSC, 10 mM EDTA pH 8.0) and Wash Buffer 3 (0.2× SSC, 10mM EDTA pH 8.0), respectively. At the end of hybridization, slides were taken out of the chamber and after removal of the cover slips they were transferred to wash vessel 1. After 10 min wash in the dark with agitation at room temperature, they were transferred to wash vessel 2, washed 10 min in the dark and the final wash was performed in vessel 3 for 10 min using Wash Buffer 3 pre-warmed to 50°C. Slides were dried by centrifugation at 15 °C for 5 min at 900 rpm and all three washing steps were repeated once again. Dried arrays were stored in the dark at 4 °C until scanning.

Step (7): scanning and analysis of microarray slides. Fluorescence scanning of each slide was performed with a microarray scanner (TECAN LS Reloaded^TM^ Microarray Scanner) for the spots corresponding to samples and markers, at 635 nm and at 532 nm, respectively. Images were analyzed by the software Array-Pro^®^ Analyzer. Subsequently, a grid of individual circles defining the name of each single dot and its position was aligned on the array and was used to correlate the fluorescence signal intensities with each dot of the array. Based on the estimation of microarray signals, which were averaged over eight individual spots, GPR files were generated and analyzed using GPR-Analyzer (GenePix^®^ *Axon Instruments*, Union City, California, USA) [43]. This software uses positive control probe intensity to normalize automatically the dataset. In fact, the signal derived from the control probes, complementary to a known amount of spiked TBP DNA fragment, provides a direct measure of hybridization efficiency. Hybridization signals were considered reliable if at least six spots in each block produced a fluorescent signal.

## 3. Results

### 3.1. Isolation and Identification of Diatom Cells from Environmental Samples

A potentially toxic diatom species was isolated from an estuarine water sample collected along the Tyrrhenian coast of central Italy (Figure 1). The original sample consisted of a mixture of several benthic pennate diatom species, which were subjected to a multi-step procedure of dilution and washing until pure cultures derived from a single cell were obtained.

**Figure 3:**
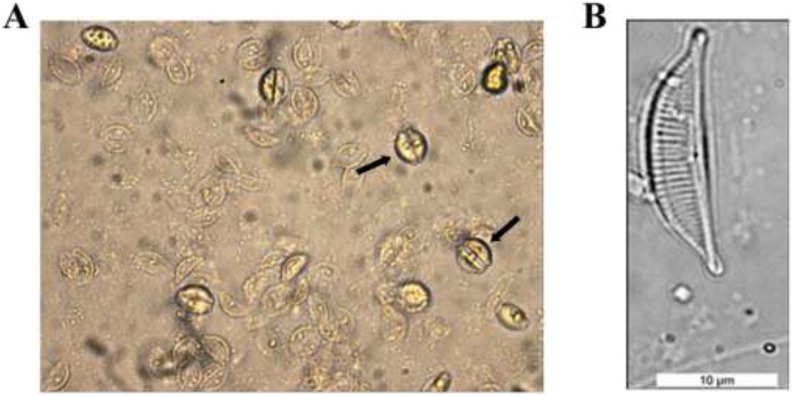
Photographs of a culture *of Amphora coffeaeformis* grown and maintained at the Laboratory of Genetics, University of Camerino (Camerino, Italy); live diatom species observed under an inverted microscope at 400× magnification are shown on panel (**A**), while the characteristic frustule of oxidized *Amphora coffeaeformis* cells, observed at 1000× magnification, is shown on panel (**B**); the arrows in panel (**A**) indicate cells undergoing cell division.

The morphological analyses of the diatom species isolated in this study was carried out at the National Institute of Health of Rome (Rome, Italy) analyzing by light microscope the features of frustules. This diatom is characterized by convex dorsal margin and straight ventral margin; in addition it presents valves lunate and semi-lanceolate, with protracted, capitate apices. The raphe is straight, and the dorsal striae radiate throughout. The size of the valves was measured for 15 frustules per field view, following standard guidelines [44]. The mean valve length was 16.76 µm (SD = 1.97) and the mean valve width was 4.52 µm (SD = 0.67). These values are within the range of length (14.2–18.6 µm) and width (4.5–7.4 µm) characteristic of this species [29]. Based on these morphological criteria, this diatom species was identified as *Amphora* *coffeaeformis* (Agardh) Kützing*.* A picture of its frustule is shown in Figure 3. The identification of the diatom species described in this study was further confirmed by molecular methods. A DNA fragment of 450 bp corresponding to a portion of the small-subunit ribosomal RNA (SSU rRNA) was amplified by PCR and sequenced. The DNA data revealed 99.1% identity with the sequence of *Amphora* *coffeaeformis* strain BA16 available in GenBank database.

This benthic diatom species is adapted to thrive in marine habitats, estuarine habitats and inland water with high conductivity. In fact, this species prefers alcalophilus habitats, tolerates elevated concentration of organically bound nitrogen and it is considered an alpha meso-eutraphentic species [45]. Thus, for optimal growth under laboratory conditions, it was necessary to optimize the medium conditions with respect to sodium chloride concentration. The pure culture of this species was propagated and used for subsequent molecular characterization.

### 3.2. Design of Universal Primers Targeting Marker Genes

In this study, we explored the use of marker genes other than ribosomal RNA for the design of species-specific oligonucleotide probes. The basic idea was to identify genes, present in all diatoms, which could be used for the design and development of short oligonucleotides that can distinguish diatoms at the species level on microarray hybridization experiments. Availability of two complete genomes of marine diatoms, namely *Thalassiosira pseudonana* (centric, radially symmetrical) [46] and *Phaeodactylum* *tricornutum* (pennate, bilaterally symmetrical) [47], provided a good starting point to look for diatom specific biomarkers. In addition, the partial or full sequences of genes of both marine and freshwater diatoms were retrieved from GenBank and were used to build the reference list of marker genes. Several genes were investigated, like fucoxanthin, myosin, translation initiation factor eIF2, translation elongation factor eEF1-a and the diatom-specific gene coding for silicic acid transporter (*SIT*). The first step consisted in the multiple alignments of the known sequences, followed by identification, for each target gene, of highly conserved regions where a candidate set of universal forward and reverse primers could be placed. After locating the oligonucleotides in the conserved segments of the alignment, primer properties were checked using Oligocalc web tool. The universal primer pair should theoretically serve to amplify a fragment of around 450 bp from the genomes of all diatoms. When the universal primer pairs targeting each of the genes under investigation were empirically tested in PCR-based assays using as template the diatom DNA bank available at the laboratory of Genetics of the University of Camerino (Camerino, Italy), the *in silico* predicted properties were experimentally confirmed only for two molecular markers of the original pool of genes, namely elongation factor 1-a (eEF1-a) and silicic acid transporter gene (*SIT*), which were used for subsequent analyses. As a result of this strategy based on sequence alignment and design, we obtained primer pair UniE-F/UniE-R and UniS-F_1_/UniS-R_1_ suitable for DNA amplification of eEF1-a and *SIT*, respectively (Table 1).

### 3.3. Design of Species-Specific Oligonucleotide Probes

**Figure 4:**
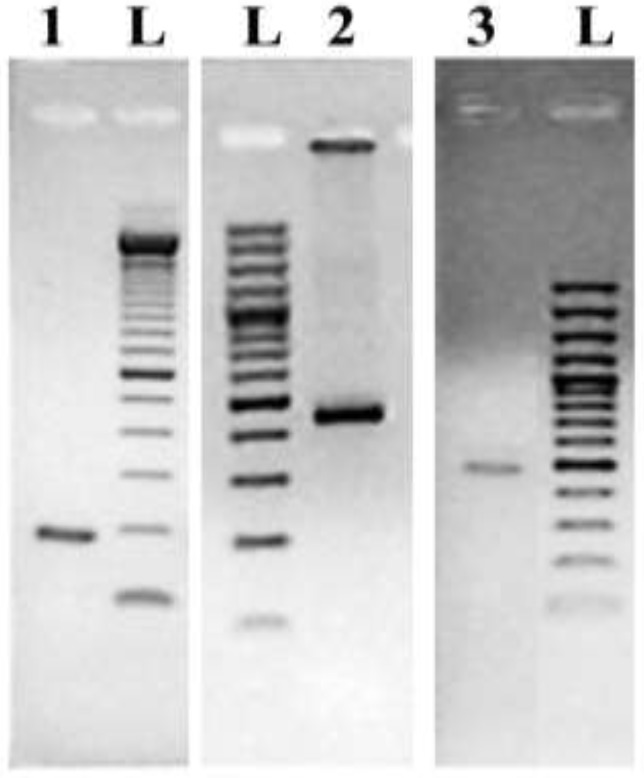
Electrophoretic separation of the PCR products obtained from amplification of *Amphora coffeaeformis* chromosomal DNA with primer pairs: (1) specific for small-subunit rRNA; (2) UniE-F and UniE-R, specific for a portion of the gene coding for the diatom elongation factor eEF1-a and (3) UniS-F_1_ and UniS-R_1_, specific for a portion of the gene coding for the diatom silicic acid transporter (*SIT*). The DNA fragment shown in lane 3 represents the amplicon after purification by agarose gel extraction. Lane L contains the 100 bp DNA ladder (Fermentas).

Universal primers targeting the two candidate marker genes were used for PCR-mediated DNA amplification of selected regions of the *A. coffeaeformis* genome. The resulting amplicons (Figure 4) were sequenced and these data were used for the design of species-specific probes.

We sought to devise a procedure suitable for the selective identification of *A. coffeaeformis*, but could also be expanded to cover all diatom species for which the DNA sequence of the two marker genes can be determined. A starting panel of 34 sequences corresponding to eEF1-a and 36 sequences corresponding to *SIT* were used for alignment, BLAST analysis and oligonucleotide probe design.

**Figure 5:**
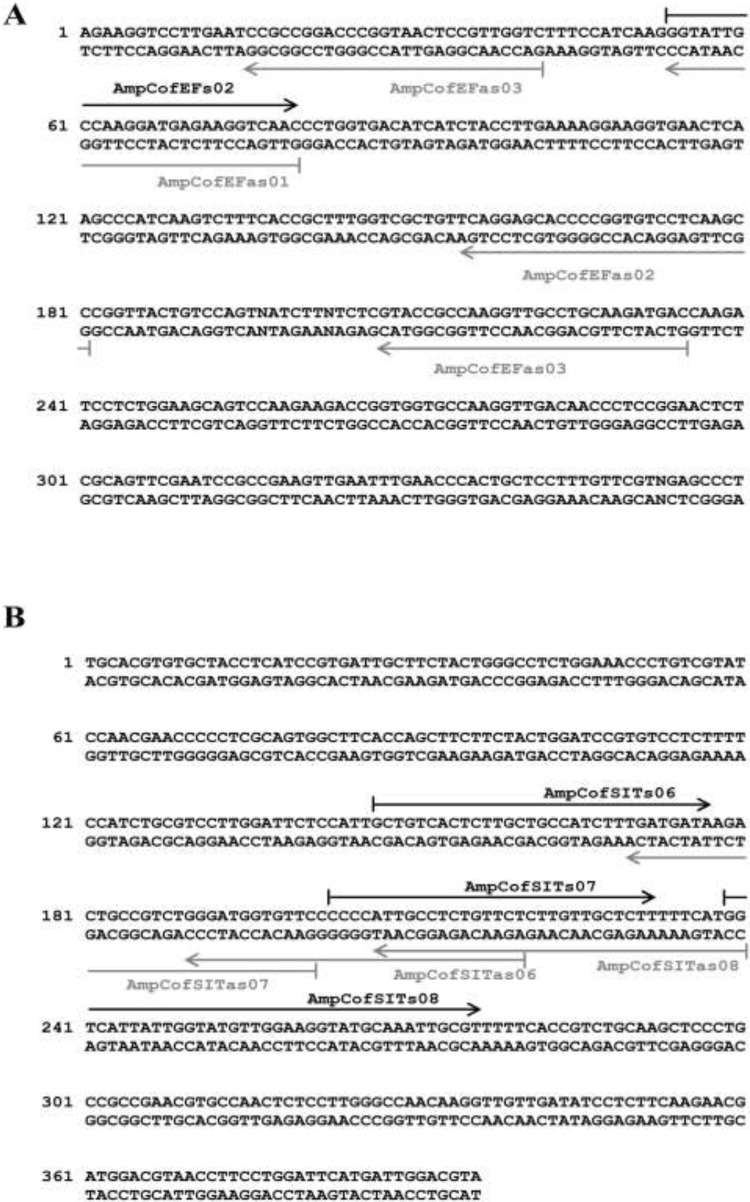
DNA sequence of the fragments of elongation factor eEF1-a (**A**) and *SIT* (**B**) obtained by PCR amplification from *Amphora coffeaeformis* chromosomal DNA. The sense and anti-sense probes specifically designed on this sequence and used for microarray analysis are indicated with black arrows and grey arrows, respectively.

The newly determined sequences of *A. coffeaeformis* amplicons were placed at the top of this panel. As a general rule, a strategy of designing at least six detection probes, three probes on sense strand and three probes on anti-sense strand was adopted to increase levels of confidence in the microarray experiments. The result of probe design activity is shown in Figure 5, where the designed oligonucleotides are displayed on the DNA sequence of both marker genes. All probes were designed within a narrow range of theoretical melting temperature (Tm = 58–64 °C) because they should all work simultaneously in the same hybridization reaction. Common parameters selected for the design of the oligonucleotide sequences were: melting temperature = 58–64 °C, length = 25–40 nt, minimum base pairs required for single primer self-dimerization = 4 bp; minimum base pair required for hairpin = 3 bp and GC content = 40%–60%.

### 3.4. Use of Microarrays for the Detection of Amphora Coffeaeformis

**Figure 6:**
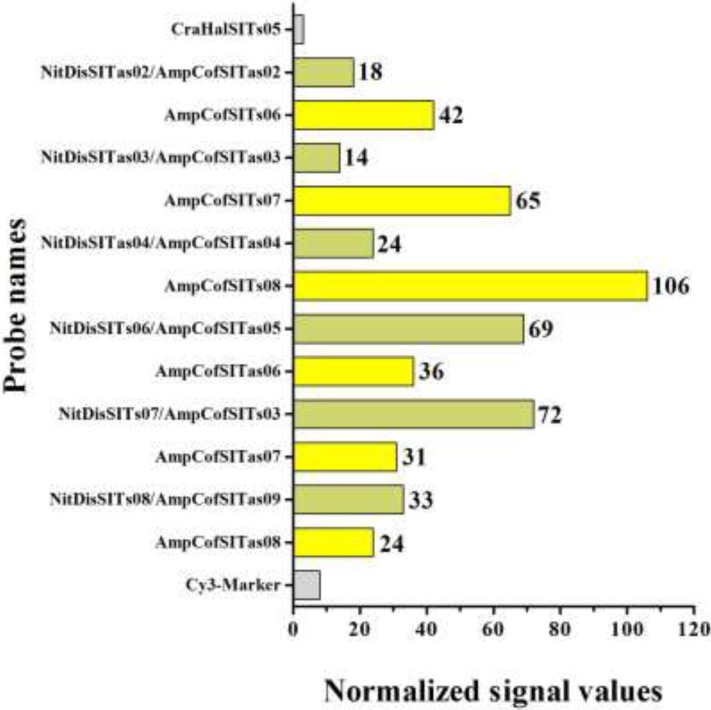
Quantification results of microarray hybridization experiments targeting *SIT* gene of *Amphora* *coffeaeformis*. On the Y-axis are reported the probes producing a fluorescent signal after hybridization: yellow bars correspond to probes displaying high specificity, green bars indicate probes with lower specificity, while gray bars correspond to probes producing false-positive signals. The fluorescence intensity signals are reported on the histogram bars.

The aim of the consortium of laboratories involved in the MicroAqua project [48] was to develop a molecular tool for the simultaneous recognition of bacteria, cyanobacteria and protozoa in freshwater environmental samples. An additional task consisted in the detection of a panel of nine diatoms that are considered diagnostic for the determination of water quality levels. To test the specificity of the oligonucleotide probes designed for *A. coffeaeformis*, the arrays developed in this study were hybridized with targets obtained by DNA amplification of eEF1-a and *SIT* with universal primers. Figure 2 shows the result of a hybridization in which the fluorescently labeled fragment of *SIT* was tested with MicroAqua-array-02. The presence on the chip of 30 oligonucleotides targeting the same gene (*SIT*) of five strictly freshwater diatoms served as control of specificity. The graph of normalized signal intensities at each spot clearly indicates the appearance of reliable and intense signals for 12 probes (Figure 6). Six probes identified by yellow bars can be considered highly specific (AmpCofSITs06, AmpCofSITs07, AmpCofSITs08, AmpCofSITas06, AmpCofSITas07 and AmpCofSITas08), because they produce a strong signal with *A. coffeaeformis SIT* target only, while six probes (NitDisSITs07/AmpCofSITs03, NitDisSITas02/AmpCofSITas02, NitDisSITas03/AmpCofSITas03, NitDisSITas04/AmpCofSITas04, NitDisSITs06/AmpCofSITas05 and NitDisSITs08/AmpCofSITas09) (green bars) have slightly lower specificity due to cross-reaction with the sequence of a *SIT* derived from a different diatom (*Nitzschia dissipata*).

A similar pattern was obtained with the target DNA of elongation factor gene. It is notable that for both marker genes the intensity of fluorescence at spots other than *A. coffeaeformis* were all near or below the threshold value. Overall, little or no cross-reactivity was recorded between the *A. coffeaeformis* labeled targets and the non-specific probes present on the chip. When labeled fragments of the marker genes obtained from nine control diatoms were hybridized with the arrays developed in this study, few cross-reactive signals were observed [48]. Based on the results of hybridization assays performed under different experimental conditions (e.g., temperature, concentration of labeled DNA), we estimate that this diagnostic tool can reliably detect a target diatom when at least 60% of the spots of the whole set of probes based on eEF1-a and *SIT* display a detectable fluorescent signal. Under these conditions, single (or even double) cross-reactive signals can be regarded as spurious and unreliable hints.

## 4. Discussion

The increasing HAB events and the production by microscopic algae of dangerous biotoxins are responsible for diseases in humans, marine life and birds with an urgent request of monitoring programs and development of rapid and efficient diagnostic techniques. A comprehensive survey has been recently done on the molecular approaches used for monitoring the HAB events as well as on the progress in the use of oligonucleotide probes for the detection of multiple species in a single assay based on hybridization techniques [49]. Species-specific probes for the identification of microorganisms usually target the ribosomal RNA sequences. Some relevant examples of successful implementation of oligonucleotide-based probes designed on the small and large subunit RNA genes are described for the diatom *Pseudo-nitzschia* [50], Dinoflagellates (e.g., *Alexandrium*) [51], fungi [52] and macroalgae [53]. In this study we describe the use of the sequence divergence of two marker genes, explored with the aim to by-pass the limitations of rRNA genes, for the development of species-specific oligonucleotides probes suitable for the detection of a diatom regarded as a potential biotoxin producer.

Among the secondary metabolites produced by diatoms there is domoic acid, a potent neurotoxin that is harmless to fish, but can be deadly to birds, marine mammals and humans that ingest contaminated fish. The precise role of this toxin and the environmental factors triggering its production are largely unknown at present. A direct link between nutrients (e.g., phosphate, nitrate, iron) or silicate limitation and synthesis of domoic acid has been hypothesized, at least in the case of *Pseudo-nitzschia* [54,55]. There is also evidence for a relationship between the presence of symbiotic bacteria, which differ in composition between toxin-producer and non toxin-producer *Pseudo-nitzschia* strains, and stimulation of the production of domoic acid [56,57].

*Amphora coffeaeformis*, a marine benthic diatom species generally found in brackish-water environments, has been reported as a producer of domoic acid [20,21]. In the present study we have used a combination of bioinformatic analyses and microarray technology to devise a novel approach for detecting specific diatoms in environmental samples. This strategy has been successfully applied to *A. coffeaeformis*, a potential toxin producer for which a surveillance tool for both human and animal health would be required. We have investigated the suitability of two unexplored genes, coding for elongation factor 1-a (eEF1-a) and silicic acid transporter (*SIT*), to distinguish diatoms at the species level. Universal primers targeting conserved regions of these marker genes were designed to selectively amplify by PCR the target DNA fragments from virtually all diatoms of interest. These universal primers were effectively used in this study to obtain amplicons and DNA sequences of the two marker genes of *A. coffeaeformis*.

Following DNA sequence alignment and clustering, probes were selected based on their ability to discriminate, *in silico*, diatom species due to sequence divergence. The designed oligonucleotide probes were then transferred to a DNA chip format suitable for hybridization experiments. The set of six oligonucleotide probes designed and tested on *A. coffeaeformis* produced a positive and very specific response. The cross-reactive signals detected with six probes (green bars in Figure 6) are frequently observed in microarray experiments and could be due to similarity of the target regions where the search activity tool located the best oligonucleotide probes. Furthermore, the hybridization conditions chosen, mainly with respect to temperature, represent a compromise and do not necessarily fit the best annealing conditions for all probes. It should be remarked that high-resolution microarrays like this can be updated as the sequences of additional marker genes of interest become available in response to ongoing research activities and entries in the databases. This would progressively lead to refined sequences of oligonucleotide probes and in turn to DNA arrays displaying enhanced sensitivity.

The application of microarray technology for identification of *Pseudo-nitzschia* spp. in the coastal waters of the northeast Pacific Ocean has recently been reported. These authors selected 307 probes targeting the internal transcribed spacer (ITS1) of the ribosomal operon of 118 *Pseudo-nitzschia* types [58]. In this assay, the PCR-amplified ITS1 region is used for hybridization to DNA microarrays, the target goal consisting of four probes for each ribotype. In line with this approach, any microarray assay dependent on preliminary DNA amplification suffers from the lack of quantitative response. However, this is just one of the inherent limitations of microarray technology, the other being the narrow dynamic range, the need for high quality biological material, the complexity of the output data and the time required for accurate data analyses [59,60]. A possible use of microarray results to obtain semi-quantitative data, might derive from implementation of S2C (Signal-to-Cell) a software specifically developed for the prediction of cell counts from microarray data. This tool allows cell count estimation via supervised learning from multi-species microarray calibration dataset. A test phase of this application is currently undergoing in our laboratory using known amounts of cells of *A. coffeaeformis* as well as of other diatoms considered good indicators of water quality levels [48].

A new approach, based on next-generation sequencing, has recently been described for the detection of freshwater diatoms [61]. The use of this technology to investigate the composition of diatom communities found in environmental samples could be a promising alternative to current detection methods and its implementation will benefit in the near future from DNA sequence data of complete freshwater diatom genomes, which are now unavailable. Expansion of diatom sequence databases may eventually lead to the application of direct high-throughput sequencing, without the bias introduced by PCR amplification of barcode genes. In the absence of genomic information regarding *A. coffeaeformis* and with the transcriptome data being available only recently [62], this work provides a novel contribution to the knowledge of two specific genes.

The microarray-based approach described in this work is by no means exhaustive and requires the support of a thorough appraisal of the response of this DNA chip to biological materials derived from complex and challenging environmental water samples. However, in the present format our data already contribute additional identification tools for diatoms, taking into account the limited repertoire of “barcode” genes described by other authors for the assessment of diatom biodiversity [63,64,65].

## 5. Conclusions

In this study, we present a strategy based on molecular methods for the detection of the diatom species *Amphora coffeaeformis*, a potential producer of the harmful neurotoxin domoic acid. The plan involved the exploration of two new marker genes, namely elongation factor eEF1-a and the diatom-specific silicic acid transporter (*SIT*) for the development of “universal primers” suitable for amplification of the target regions from chromosomal DNA of *A. coffeaeformis* (as well as other diatoms species). Next, the sequences of these two marker genes were used as a source of genetic diversity for the design and application of species-specific oligonucleotide probes. When tested in microarray hybridization experiments, these molecular probes displayed high ability to recognize *A. coffeaeformis* target genes*.* Although the molecular tool described in this work is preliminary and subject to refinement and amendment after a test phase with complex environmental water samples, the DNA chip technology in the present layout proves suitable for monitoring the presence of this potential biotoxin producer diatom.

## Supplementary Files

Supplementary File 1
