# Supplementary material for: Molecular Detection of a Potentially Toxic Diatom Species
Source: Int J Environ Res Public Health. 2015 May 6;12(5):4921–41. doi: 10.3390/ijerph120504921 (PMC4454946; doi:10.3390/ijerph120504921)
Supplement: Supplementary File 1 [file ijerph-12-04921-s001.pdf]

Molecular Detection of a Potentially Toxic Diatom Species

Figure S1. List of oligonucleotides spotted on the MicroAqua-array-01.

MicroAqua-array-01 Probe Name
POSITIVE_25_dT
AMP_COF_P29 (Fo)
AMP_COF_P29 (Re)
AMP_COF_P22 (Fo)
AMP_COF_P25 (Fo)
AMP_COF_P25 (Re)
AMP_COF_P20 (Fo)
AMP_COF_P20 (Re)
AMP_COF_P21 (Fo)
AMP_COF_P21 (Re)
ACH_FLE_Tag #16 (Fo)
ACH_FLE_Tag #16 (Re)
ACH_FLE_Tag #5 (Fo)
ACH_FLE_Tag #5 (Re)
CAL_BAC_Tag #23 (Fo)
CAL_BAC_Tag #23 (Re)
GOM_ANG_Tag #8 (Fo)
GOM_ANG_Tag #8 (Re)
MEL_VAR_Tag #41 (Fo)
MEL_VAR_Tag #41 (Re)
MEL_VAR_Tag #54 (Fo)
MEL_VAR_Tag #54 (Re)
MEL_VAR_Tag #56 (Fo)
MEL_VAR_Tag #56 (Re)
NIT_COM_Tag #28 (Fo)
NIT_COM_Tag #28 (Re)
NIT_COM_Tag #52 (Fo)
NIT_COM_Tag #52 (Re)
NIT_COM_Tag #55 (Fo)
NIT_COM_Tag #55 (Re)
NIT_DIS_P24 (Fo)
NIT_DIS_P24 (Re)
NIT_DIS_P27 (Fo)
NIT_DIS_P27 (Re)
NIT_SIG_P21 (Fo)
NIT_SIG_P21 (Re)
NIT_SIG_P26 (Fo)
NIT_SIG_P26 (Re)
NIT_VIT_Tag #7 (Fo)
NIT_VIT_Tag #7 (Re)
NIT_VIT_Tag #65 (Fo)
NIT_VIT_Tag #65 (Re)
NIT_VIT_Tag #67 (Fo)
NIT_VIT_Tag #67 (Re)
NIT_VIT_Tag #19 (Fo)
NIT_VIT_Tag #19 (Re)
PLE_LAE_Tag #2 (Fo)
PLE_LAE_Tag #2 (Re)
SUR_OVA_Tag #36 (Fo)
SUR_OVA_Tag #36 (Re)
SUR_OVA_Tag #46 (Fo)
SUR_OVA_Tag #46 (Re)
SUR_OVA_Tag #7 (Fo)
SUR_OVA_Tag #7 (Re)
SUR_OVA_Tag_probe#36
CAL_BAC_Tag_probe#23
GOM_ANG_Tag_probe#20
MEL_VAR_Tag_probe#56
SUR_OVA_Tag_probe#46
ACH_FLE_Tag_probe#11
MEL_VAR_Tag_probe#41
NIT_VIT_Tag_probe#67
NIT_VIT_Tag_probe#7
NIT_VIT_Tag_probe#65
SUR_OVA_Tag_probe#7
ACH_FLE_Tag_probe#16
NIT_VIT_Tag_probe#19
GOM_ANG_Tag_probe#8
NIT_COM_Tag_probe#28
ACH_FLE_Tag_probe#5
MEL_VAR_Tag_probe#54
NIT_COM_Tag_probe#55
AMP_COF_P20
AMP_COF_21
AMP_COF_22
AMP_COF_25
AMP_COF_29
NIT_SIG_P21
NIT_SIG_P26
AmpCofEFs01
AmpCofEFs02
AmpCofEFs03
AmpCofEFas01
AmpCofEFas02
AmpCofEFas03
CraHalEFs01
CraHalEFs02
CraHalEFs03
CraHalEFas01
CraHalEFas02
CraHalEFas03
CycMenEFs01
CycMenEFs02
CycMenEFs03
CycMenEFs04
CycMenEFs05
CycMenEFas01
MayPerEFs01
MayPerEFs02
MayPerEFs03
MayPerEFas01
MayPerEFas02
MayPerEFas03
NavVenEFs01
NavVenEFs02
NavVenEFs03
NavVenEFas01
NavVenEFas02
NavVenEFas03
NitDisEFs01
NitDisEFs02
NitDisEFs03
NitDisEFs04
NitDisEFs05
NitDisEFas01
NitPalEFs01
NitPalEFs02
NitPalEFs03
NitPalEFs04
NitPalEFs05
NitPalEFas01
SurAngEFs01
SurAngEFs02
SurAngEFs03
SurAngEFas01
SurAngEFas02
SurAngEFas03
AmpCofSITs01
AmpCofSITs02
AmpCofSITs03
AmpCofSITs04
AmpCofSITs05
AmpCofSITas01
CraHalSITs01
CraHalSITs02
CraHalSITs03
CraHalSITas01
CraHalSITas02
CraHalSITas03
EolMinSITs01
EolMinSITs02
EolMinSITs03
EolMinSITs04
EolMinSITas01
EolMinSITas02
MayPerSITs01
MayPerSITs02
MayPerSITs03
MayPerSITs04
MayPerSITas01
MayPerSITas02
NavVenSITs01
NavVenSITs02
NavVenSITs03
NavVenSITs04
NavVenSITas01
NavVenSITas02
NitDisSITs01
NitDisSITs02
NitDisSITs03
NitDisSITs04
NitDisSITs05
NitDisSITas01
NitPalSITs01
NitPalSITs02
NitPalSITs03
NitPalSITs04
NitPalSITs05
NitPalSITs06
SurAngSITs01
SurAngSITs02
SurAngSITs03
SurAngSITs04
SurAngSITs05
SurAngSITas01

Figure S2. List of oligonucleotides spotted on the MicroAqua-array-02.

MicroAqua-array-02 Probe Name
Cy3-Marker
Buffer
Empty
AmpCofTag7F
AmpCofTag25
NitPalEFas02/NavVenEFas07
SurAngEFas03/AmpCofEFas10
OrRaphexNaviculalesSITs02
AmpCofSITas08
ClBacNavSSEFs09
NavVenSITas07
NitDisSITs08/AmpCofSITas09
SurAngEFas07
Lambda
AmpCofP20Tag41F
NitVitTag7R
NavVenEFas05/SurAngEFas02/ AmpCofEFas09
MayPerEFas03/NitDisEFas14
EolMinSITas02
AmpCofSITas07
ClBacNavSSEFs08
NavVenSITas06
NitDisSITs07/AmpCofSITs03
SurAngEFas05/AchMinEFas05/ NitPalEFas10
POSITIVE_25_dT_12.5 ï¿½M
NitDisTag46R
AmpCofTagP29
NitDisEFas04/CraHalEFas05
NavVenEFas05/SurAngEFas02/ AmpCofEFas09
NitVitSIT01
AmpCofSITas06
ClBacNavSSEFs03
NavVenSITas05
NitDisSITs06/AmpCofSITas05
SurAngEFas04/AchMinEFas04/ NitPalEFasS09
POSITIVE_25_dT_25 ï¿½M
CraHalTag23R
AmpCofP20Tag221R
CraHalEFs02/NavVenEFs07
MayPerEFas02/NitDisEFas13
EolMinSITs02
AmpCofSITs08
ClBacNavSSEFs012
NavVenSITs05
NitDisSITas04/AmpCofSITas04
SurAngEFs06
POSITIVE_25_dT_50 ï¿½M
AmpCofTag46F
NavVenTag55
NavVenEFas03
SurAngEFs01/NitDisEFs16/ NavVenEFas01
AmpCofTag10
AmpCofSITs07
ClBacNavSSEFs011
NavVenSITs04/CraHalSITs02
NitDisSITas03/AmpCofSITas03
SurAngEFs05
DunGS05_25_dT_dT_12.5 ï¿½M
NitDisTag23F
NitVitTag27F
AmpCofEFas03
CycMenEFs03
EolMinSITas01
AmpCofSITs06
ClBacNavSSEFs010
NavVenSITs03/CraHalSITs01
NitDisSITas02/AmpCofSITas02
SurAngEFs04
DunGS05_25_dT_dT_25 ï¿½M
NitPalTag36F
NitVitTag7
NavVenEFas02/NitDisEFas15
NitPalEFas01/NavVenEFas08
NitPalSITas11
ClBacNavSSEFs06
CraHalSITas06
OrNavNavSSEFs02
NitDisEFas08/SurAngEFas06/ AchMinEFas02/MayPerEFas06
NitPalEFas08
ClBacNavSSEFs03
DunGS05_25_dT_dT_50 ï¿½M
AmpCofTag5R
AmpCofEFas02
CycMenEFs02
NavVenSITas10
NavVenSITs01/CraHalSITas08
CrahalSITas05
OrNavNavSSEFs01
NitDisEFas07/SurAngEFs01/ AchMinEFas03/MayPerEFas05
NitPalEFas07
DunGS02_25_dT_dT_12.5 ï¿½M
NitDisTag67R
NitPalTag23
NavVenEFas09/AmpCofEFas01/ CraHalEFas01
NitPalEFas05/AmpCofEFas11
OrRaphSITs01
ClBacNavSSEFs05
CraHalSITas04
ClBacNavSSEFs013
NitDisEFas06/AchMinEFas01/ MayPerEFas04
NitPalEFas06
ClBacNavSSEFs02
DunGS02_25_dT_dT_25 ï¿½M
AmpCofP20F
AmpCofTag
CycMenEFs01
NavVenSITas09
CraHalSITs06
OrNavNavSSEFas03/NitPalEFs07
NitDisEFs04/MayPerEFs03
NitPalEFs04
DunGS02_25_dT_dT_50 ï¿½M
NitDisTag67F
NitDisTag7R
SurAngEFs01/NitDisEFs16/ NavVenEFas01
NitPalEFas04/NavVenEFas04
AmpCofTag09
ClBacNavSSEFs04
CraHalSITs05
OrNavNavSSEFas02/NitPalEFs06
NitDisEFs03/MayPerEFs02
NitPalEFs03
ClBacNavSSEFs01
euk 1209
AmpCofTag29R
AmpCofP29Tag54R
AmpCof29Tag
CraHalSITs04
OrNavNavSSEFas01/NitPalEFs05
NitDisEFs02/MayPerEFs01
NitPalEFs02
NavVenSITs02/CraHalSITs09
euk 328

Table S1. Sequences of oligonucleotide probes spotted on microarray slides, recognizing *Amphora coffeaeformis* marker genes.

Marker Gene	Probe Name		Probe Sequence 5' → 3'
Elongation Factor gene (eEF1-a)	Sense strand	AmpCofEFs01	GTTGACCTTCTCATCCTTGGCAATACC
		AmpCofEFs02	GGTATTGCCAAGGATGAGAAGGTCAAC
		AmpCofEFs03	GACCAACGGAGTTACCGGGTCCGGCGG
	Antisense strand	AmpCofEFas01	GGTTGACCTTCTCATCCTTGGCAATACC
		AmpCofEFas02	GGCTTGAGGACACCGGGGTGCTCCTGA
		AmpCofEFas03	GTCATCTTGCAGGCAACCTTGGCGGTAC
Silicic Acid Transporter gene (*SIT*)	Sense strand	AmpCofSITs06	GCTGTCACTCTTGCTGCCATCTTTGATGATA
		AmpCofSITs07	CCCCATTGCCTCTGTTCTCTTGTTGCTCTT
		AmpCofSITs08	GGTCATTATTGGTATGTTGGAAGGTATGCAAATTGCGT
		NitDisSITs07/AmpCofSITs03	AATCTCTTCTTCTGGGGACGATGCCTCTTCTCT
	Antisense strand	AmpCofSITas06	AGAACAGAGGCAATGGGGGGAACACCATCCC
		AmpCofSITas07	GAACACCATCCCAGACGGCAGTCTTATCATCA
		AmpCofSITas08	CCATGAAAAAGAGCAACAAGAGAACAGAGGCAAT
		NitDisSITas02/AmpCofSITas02	GCAAAAGCACACGACGGCGAAAGAGAAG
		NitDisSITas03/AmpCofSITas03	AGAAGAAGAGATTGGCCACGGCACTGC
		NitDisSITas04/AmpCofSITas04	AAGCACAATTCCCTTGGGGATTCCCTCCCA
		NitDisSITs06/AmpCofSITas05	CACCATGAGCACTCTCTTCAACGAACAAACCAC
		NitDisSITs08/AmpCofSITas09	GCTGTTGCCAAGATCCGCGAGTCGGAA

Colour code of oligonucleotide probes:

Species-specific probe
Probe recognizing two species

© 2015 by the authors; licensee MDPI, Basel, Switzerland. This article is an open access article distributed under the terms and conditions of the Creative Commons Attribution license (http://creativecommons.org/licenses/by/4.0/).
